# Private Nurses and the Hours of Employment Bill

**Published:** 1921-01-08

**Authors:** Geraldine Bremner

**Affiliations:** 63 Wimpole Street, W. 1


*To the Editor of* THE HOSPITAL.

January 4, 1921.

### Private Nurses and the Hours of Employment Bill.

THE EFFECT OF A "SPECIAL ORDER."

*To the Editor of* THE HOSPITAL.

SIR,—I wish to thank all those who have so kindly responded to my request to send me their views as to the advisability of the inclusion of private nurses in a State Bill for the regulation of nurses' hours. Many have replied, but not by any means all, whose opinion is required. My fellow-sisters will understand that if they wish me to help them they must help me. No Member of Parliament can be of any use to his constituents unless he knows their views. One or two sisters ask me that their names may be kept confidential. This is thoroughly understood. It is the opinions of the majority I want, that I may honestly and truly as their representative voice their wishes.

I hope that sisters when thinking of legislation fully understand the difference between the inclusion in the Hours of Employment Bill as now drafted and inclusion in a "Special Order" under the Bill. The reason for a "Special Order" is that the peculiar needs of any trade or profession shall be especially provided for. For instance, in the scheme which the College drew up the private nurse is allowed to arrange the hours of duty as the needs of the patient determine. During the critical period the nurse would be on duty as many hours as she deems necessary. Her safeguard, however, would be that during the convalescent period she would work fewer hours, so that over a certain period, say, two or three weeks, she did not exceed the number of hours prescribed by the "special order." Whether it should be a forty-eight or a fifty-six hours' week seems immaterial just now; that could be settled in conference with all nurse societies. The chief point is the principle of the State Regulation of Hours in a Special Order.—Yours faithfully, GERALDINE BREMNER.

63 Wimpole Street, W. 1. January 4, 1921.

